# Identification and characteristics of vaccine refusers

**DOI:** 10.1186/1471-2431-9-18

**Published:** 2009-03-05

**Authors:** Feifei Wei, John P Mullooly, Mike Goodman, Maribet C McCarty, Ann M Hanson, Bradley Crane, James D Nordin

**Affiliations:** 1 HealthPartners Research Foundation, Minneapolis, MN, USA; 2 Center for Health Research, Kaiser Permanente Northwest, Portland, OR, USA; 3 Xcenda, Woodlands Parkway, Palm Harbor, FL, USA

## Abstract

**Background:**

This study evaluated the utility of immunization registries in identifying vaccine refusals among children. Among refusers, we studied their socioeconomic characteristics and health care utilization patterns.

**Methods:**

Medical records were reviewed to validate refusal status in the immunization registries of two health plans. Racial, education, and income characteristics of children claiming refusal were collected based on the census tract of each child. Health care utilization was identified using both electronic medical record and insurance claims. Within the immunization registries of two HMOs in the study, some providers use refusal and medical contraindication interchangeably, and some providers tend to always use "ever refusal." Therefore, we combined medical contraindication and refusal together and treated them all as "refusal" in this study.

**Results:**

The immunization registry, compared to chart review, had negative predictive values of 85–92% and 90–97% for 2- and 6-year olds, and positive predictive values of only 52–74% and 59–62% to identify vaccine refusals. Refusers were more likely to reside in well-educated, higher income areas than non-refusers. Refusers had not opted out of health care system and continued, although less frequently for the age 2 and under group, to use services.

**Conclusion:**

Without enhancements to immunization registries, identifying children with immunization refusal would be time consuming. Since communities where refusers live are well educated, interventions should target these communities to communicate vaccine adverse events and consequences of vaccine preventable diseases.

## Background

The decision to voluntarily forgo immunization exposes both the children whose parents refused vaccination and others in the community to the risks of vaccine-preventable disease.[1]

Mandatory vaccination for school entry, as required in all states, is one effective way the United States has chosen to control vaccine preventable diseases. Although this policy exists in all states, exemptions are still allowed. The reasons for parents to refuse to have their children vaccinated ranged from religious beliefs to, increasingly, a fear of adverse reactions to certain vaccinations. A recent study showed that the most common reason stated for refusal (190 [69%] of 277) was concern that the vaccines might cause harm.[2] Parents who refused to vaccinate their children were significantly more likely than parents of vaccinated children to report low perceived vaccine safety and efficacy, a low level of trust in the government, and low perceived susceptibility to and severity of vaccine-preventable diseases. Parents who refused specific or all vaccinations for their children were significantly less likely to report confidence in medical, public health, and government sources for vaccine information and were more likely to report confidence in alternative medicine professionals than parents of vaccinated children.

Health plans have the rare ability to document parental vaccine refusal for a large number of children of all ages. This study took advantage of two health plans' ability to track immunization status, enrollment, and medical encounters to examine immunization refusals.

The purpose of this study was to understand methods for identifying refusers and, among those refusers, to describe their social characteristics and health care utilization patterns. To accomplish the first aim we sought to evaluate the utility of HMO immunization registries in identifying vaccine refusals. The knowledge of HMO immunization registry completeness and the accuracy of social and medical care correlates of vaccine refusals will aid in the development of interventions to improve immunization compliance and long-term follow-up of refusers. Specifically, the study objectives were firstly to assess levels of agreement between automated immunization registries and medical chart abstractions of vaccine refusals. Secondly, the study describes differences in social and economic characteristics (at the census tract level) between children in the age group of 2 to 6 years old who were not vaccinated because of parental refusal and children who were vaccinated. Thirdly, we described patterns of health care utilization (well-child, outpatient, prescriptions, ER and hospital visits) for children with a refusal of vaccination from birth to ages two and six.

## Methods

The study protocol was approved by the institutional review boards at the two participating sites and the Centers for Disease Control and Prevention.

### Study population

The study population was composed of children aged 0 through 6 years old enrolled in two large health plans participating in the CDC-sponsored Vaccine Safety Datalink (VSD) Project. Children who were enrolled in HealthPartners (HP) from 1997–2001 or Kaiser Permanente Northwest (NWK) from 1998–2001 were included in the study. These two HMOs were chosen because of geographic location and nationwide affiliation. One located on the west coast, and the other in the Midwest. One is part of a national HMO, and the other is a regional HMO.

HP documented immunization refusal beginning in 1997 as either "medical contraindication" or "parent/patient refusal." NWK documented immunization refusal in its vaccination database beginning in 1998 as either "Medical Contraindication to Immunizations" or "Patient/Parent Refusal of Immunizations." Medical contraindication indicated in the registry could be, for example, that the child had a minor fever at time of visit, and the parent chose not to let the child get vaccinated at that time. In addition, some providers use refusal and medical contraindication interchangeably, and some providers tend to always use "ever refusal." Therefore, we combine medical contraindication and refusal together and treat them all as "refusal" in this study.

### Eligibility Criteria

The study used the VSD 2002 annual database. Children born between 1994 and 2001 for HP and children born between 1993 and 2001 for NWK were eligible for the study. Henceforth, the two HMOs will be referred to as HMO A and HMO B due to an IRB requirement that prohibits identifying the specific data that came from each HMO.

Clinics at both HMOs maintain vaccination histories in an immunization registry module of electronic medical records for all patients seen at clinics. Existing patients who visit clinics have had their historical immunization data verified and recorded. As new patients come into the system, immunization histories are entered directly into electronic medical record system by the provider. A copy is made of the outside record and scanned into the system to allow for quality assurance. The refusal information is entered in the immunization registry at the point of service. The VSD 2002 annual database used in this study included immunization data from both the immunization registry and the billing system.

Study inclusion criteria are shown in Table 1. The "Age 2 and under" group and "age 6" group were chosen based on the inherent nature of immunization schedule. There is an intense series of immunizations between birth and the age of 18 months, where a number of vaccinations must be given within the short time span of a few months. After these initial vaccinations, no vaccinations are routinely recommended between ages 2 and 4. After age 4, recommended immunizations could be taken within a 2-year time span between the ages of 4 and 6. Due to the requirement of the School Immunization Laws, a health care provider of a 6-year old likely has the child's most up-to-date immunization status. Therefore, this study had a continuous enrollment criteria for the "Age 2 and under" group, but only a point-enrollment requirement at age 6 for the "age 6" group. Since some refusals may be due to a temporary medical condition (such as a headache), the child may receive a vaccination at a later visit. Therefore, some of ever-refuse children, did achieve up-to-date immunization status at the evaluation age specified in Table 1.

**Table 1:** "Age 2 and under" and "Age 6" eligibility and up-to-date status criteria

**Age 2 and under Group**			
**Age by 12/31/01**	**Enrollment criteria^a^**	**Up-to-date status evaluated at**	**Up-to-date status criteria^b^**

>= 24 months old	Birth to 15 months of age	At age 2	4 DtaP, 3 IPV, 1 MMR, 3 Hib, 3 HBV
>= 18 and < 24 months	Birth to 15 months of age	at 12/31/2001	4 DtaP, 3 IPV, 1 MMR, 3 Hib, 3 HBV
>= 15 and < 18 months	Birth to 15 months of age	at 12/31/2001	3 DtaP, 2 IPV, 1 MMR, 3 Hib, 2 HBV
>= 6 and < 15 months	Birth to 12/31/2001	at 12/31/2001	3 DtaP, 2 IPV, 2 Hib, 2 HBV
>= 4 and < 6 months	Birth to 12/31/2001	at 12/31/2001	2 DtaP, 2 IPV, 2 Hib, 2 HBV
< 4 months	Not eligible for "age 2 and under" sub-study		

**Age 6 Group**			

**Age by 12/31/01**	**Enrollment criteria^a^**	**Up-to-date status evaluated at**	**Up-to-date status criteria^b^**

>= 6 years old	Enrolled at age 6	At age 6	5 DtaP/DTP, 4 IPV/OPV, 2 MMR, 3 Hib, 3 HBV
< 6 years old	Not eligible for "age 6" sub-study		

^a^Allows 32 days gaps at the beginning, at the end and in-between.

^b^DtaP: Diphtheria, tetanus, and pertussis vaccine; IPV: Inactivated Polio Vaccine; MMR: Measles, Mumps & Rubella Vaccine;

Hib: Haemophilus influenzae type b; HBV: Hepatitis B Virus

Children age 15 months and older on December 31, 2001 who were continuously enrolled (allowing 32 days gaps) in the health plan from birth to 15 months of age were included in the "age 2 and under" group. Children of age equal or greater than 4 months and less than 15 months old at 12/31/2001 who were continuously enrolled in the health plan from birth to December 31, 2001 were also included in the "age 2 and under" study population. Children of age equal or more than 6 years old who enrolled in the health plan at time of age 6 were included in the "age 6" group. Children in the "Age 2 and under" group were included to study health care utilization in the first 24 months, and children in the "Age 6 and under" group were included to compare health care utilization in the first 6 years between ever-refusers versus never-refusers.

### Chart review

Chart reviews were performed on a random sample to validate immunization status and refusal from immunization information to collect information on date and type of vaccination given, and the reason not given, if a vaccination was mentioned but not given during the visit. The sample selection mechanism is shown in Table 2. "Age 2 and under" group was chart reviewed from birth to age 2 or 12/31/2001, whichever occurred first. "Age 6" group was chart reviewed from birth to age 6 1/2 or 12/31/2001, whichever occurred first. The chart review scheme was shown in Table 2. If there were sufficient subjects eligible, we reviewed a 30% random sample in cells involving refusers. If there were not enough eligible subjects in cells involving refusers, we chart reviewed all available charts. For the non-refusers, we over sampled those who were not up-to-date within the restrictions of the budget at each participating site (Table 2).

**Table 2:** Study population

**Age 2 and under Group**				
	HMO A		HMO B	

	# Eligible	# Chart reviewed	# Eligible	# Chart reviewed
UTD^a^/Ever Refuse	19	15 (78.9%)	255	74 (29.0%)
UTD/Never Refuse	10,446	100 (1%)	18,335	71 (0.4%)
Not UTD/Ever Refuse	77	68 (88.3%)	202	66(32.7%)
Not UTD/Never Refuse	3,715	342 (9.2%)	6,097	104 (1.7%)
**Total**	**14,257**	**525 (3.68%)**	**24,889**	**315 (1.27%)**

**Age 6 Group**				

	HMO A		HMO B	

UTD/Ever Refuse	8	8 (100%)	31	28 (90.3%)
UTD/Never Refuse	3,128	30 (0.96%)	7,789	63 (0.8%)
Not UTD/Ever Refuse	25	24 (96%)	34	31 (91.2%)
Not UTD/Never Refuse	1,960	153 (7.8%)	5,305	72 (1.4%)
**Total**	**5,121**	**215 (4.20%)**	**13,159**	**194 (1.47%)**

^a^UTD: Up-to-date immunization status

Chart abstraction was completed by trained reviewers with years of experience in record abstraction. Training consisted of approximately 4 hours of classroom review of study objectives, vaccination information, and review of 2 sample charts. Abstractors then completed test charts to assure uniformity of review by all abstractors.

### Electronic Data collection

VSD data files were queried for study subjects who met study inclusion criteria. These data included information on enrollment, refusal status, vaccinations received, and dates of vaccine receipt. VSD data files were also used to identify outpatient, emergency department and urgent care visits, and hospitalizations.

### Variable definition

A child was classified as a refuser if he/she had a record of a vaccine refusal either in the medical chart or in the immunization registry. A child's immunization up-to-date status was determined based on his immunization data gathered from immunization registry and supplemented by the medical record according to criteria in Table 1.

In order to avoid alienating patients by collecting potentially sensitive information,[3] the participating HMOs as well as most providers throughout country, do not routinely collect demographic variables of children or their parents. The study, therefore, identified the census block group in which each participant lived by geocoding his/her residential address as of December, 2001. [4-24] The census block group was defined as a child's "community." For each child, the study collected census information on race (white/non-white), proportion of individuals older than 25 years of age, educational status, and household income. In accordance with the census definition, a child was below poverty level if his/her total household income was less than the poverty threshold specified for the applicable family size, age of householder, and number of related children under 18.[25]

We identified four conditions that were likely to occur frequently and lead to higher medical care utilization: otitis media and other ear disorders (ICD-9 diagnosis codes = 381, 382, 388); respiratory, including Asthma (493, 786, 466); skin, including Dermatitis (691, 692); seizure (345, 780.3) diagnoses.[26]

Outpatient visits included both regular and urgent care visits: well child visit (ICD-9 diagnosis codes of V20 and V70), illness visit (000–799), and injury visit (800–999). Well child visits were defined as outpatient visits with ICD-9 diagnosis codes of V20 and V70. Only non-birth hospital stays were counted toward inpatient visits. Drug prescriptions studied were antibiotics, asthma, and seizure medications, which were commonly prescribed to infants and children.

### Analysis

The study collected refusal information found in the medical record and in the HMO immunization registry of our chart review sample. If the child had any type of refusal (either parent refusal or medical contraindication) during the study period for any vaccine they were marked as having a refusal.

Univariate statistical comparisons were made with chi-square test for categorical variables and t-tests for continuous variables. The study used logistic regressions, Poisson regression models and general linear models to estimate adjusted odds ratios as follows: we compared ever/never use of well-child care, outpatient, emergency room and inpatient care, and select prescriptions between refusers and non-refusers (both up-to date and not up-to-date for immunization), employing logistic regressions that controlled for HMO site, patient's gender, immunization up-to-date status, high use medical conditions, Medicaid status in 2001, and the child's community race/SES variables. Differences in quantitative community characteristics and rates of health care utilization were assessed using Poisson regression models and general linear models with or without log transformations that adjusted for the same set of variables.

## Results and discussion

There are total of 14,257 children from HMO A and 24,889 children aged 2 years old or younger from HMO B, and 5,121 children from HMO A and 13,159 children aged 6 from HMO B who were eligible for the study. A total 830 children aged 2 years old and under and 409 children aged 6 were selected for chart review.

Negative predictive values of electronic data compared to chart review on immunization refusal status were 92% and 90% for 2 years old and under and 6-year olds for HMO A, and 85% and 97% for HMO B, respectively. Positive predictive values were only 52% and 62% for HMO A and 74% and 59% for HMO B, using a definition of ever refusal (any refusal for any vaccination) (Table 3). Only between 52% and 74% of refusals documented in the immunization registry were confirmed by text in the electronic medical record. Due to poor positive predictive values, this study cannot provide a population estimate of the proportion of children with immunization refusal in the study population. Closer review of the 21 cases from the 6-year old sample with a refusal in both chart review and HMO immunization registry revealed multiple mismatches with respect to date of refusal and vaccine refused.

**Table 3:** Agreement between chart review and HMO immunization registry on ever refusal status

**Agreement %** **(95% CI)**	HMO A	
	**Two years old and under** ***n *= 525**	**Six years old** ***n *= 215**

Sensitivity	58.5% (47.1%, 69.2%)	52.5% (36.3%, 68.2%)
Specificity	89.8% (86.6%, 92.4%)	92.6% (87.4%, 95.8%)
Positive predictive value	51.6% (41.1%, 62.0%)	**61.8%^#^(43.6%, 77.3%)**
Negative Predict value	92.1% (89.1%, 94.4%)	89.5% (83.9%, 93.4%)
Kappa	45.9% (35.8%, 56.0%)	47.8% (32.4%, 63.3%)

	HMO B	

	**Two years old and under *n *= 315**	**Six years old *n *= 194**

Sensitivity	79.8% (71.7%, 86.2%)	**89.7%^#^(74.8%, 96.7%)**
Specificity	80.1% (73.5%, 85.5%)	84.5% (77.6%, 89.6%)
Positive predictive value	73.6% (65.3%, 80.5%)	**59.3%^#^(45.8%, 71.7%)**
Negative Predict value	85.1% (78.8%, 89.9%)	**97.0%^#^(92.1%, 99.1%)**
Kappa	59.2% (50.2%, 68.2%)	62.3% (50.0%, 74.6%)

^# ^P-value < .05

Since the agreement between HMO immunization registry and chart reviews on immunization refusal status was poor the following analyses were restricted to the chart review sample only (*n *= 1,249, Table 2).

Table 4 shows socio-economic characteristics of refuser and non-refuser communities, adjusting for up-to-date immunization status, HMO site, gender, Medicaid status, and select high use medical conditions. Compared to communities where non-refusers lived, refusers' communities had higher average household incomes for both age 2 and under (P-value = 0.04) and age 6 (P-value = 0.03) chart review samples. In the age 6 chart review sample, there were fewer households below the poverty income level (P-value = 0.04) and fewer adults without college degree (P-value = 0.01) among refusers' communities.

**Table 4:** Demographic characteristics of children communities of the chart review sample

**Adjusted^a ^Odds Ratio of Ever Refuse vs. Never Refuse**	**Age 2 and under Group**	**Age 6 Group**
% of Pop. with Income < Poverty Level^b^	0.91 (0.80, 1.04)	**0.80** **(0.64, 0.99)**^#^
% of Pop. with age >= 25 yrs and < 9 yrs of Education^b^	0.87 (0.74, 1.005)	**0.69** **(0.51, 0.92)**^#^
Non-white% > white% *versus *White% > Non-white%^c^	1.02 (0.51, 2.02)	0.262 (0.06, 1.15)

**Adjusted Mean Difference of Ever Refuse vs. Never Refuse**		

Average household income^d^	**2,820** **(156, 5,485)**^#^	**4,439** **(469, 8,409)**^#^

^a ^Adjusted for HMO, up-to-date immunization status, gender, Medicaid status, and select high use medical conditions.

^b ^General linear model with log transformation was used.

^c^Logistic regression model was used.

^d ^General linear model without log transformation was used.

^# ^P-value < .05

Only one of 236 (0.4%) refusers in the age 2 and under group had never been seen in an outpatient setting. Four (1.7%) refusers in the age 2 and under group and eight (8.7%) refusers in the age 6 group had no well-child visit. Since enrollment criteria for the age 6 group required only that a child was enrolled at a participating HMO at age 6, a child in the age 6 group may have been seen at an outpatient clinic or had a well-child visit when he/she was not enrolled at a participating HMO.

Table 5 shows adjusted odds ratios of ever taking antibiotics, asthma, or seizure medications, being admitted to a hospital, or visiting an emergency room between refusers and non-refusers. Covariates included were up-to date immunization status, HMO site, gender, Medicaid status, and select high use medical conditions. The majority of age-6 group children took antibiotics, asthma, or seizure medications. Compared with non-refusers, a higher percentage of refusers took antibiotics, asthma, or seizure medications in the age 6 group (93.48% versus 77.90%, P-value = 0.0003).

**Table 5:** Adjusted^a ^odds ratios of health care utilization of children in the chart review sample

	**Age 2 and under Group**		**Age 6 Group**	
	**Ever Refuse vs.** **Never Refuse**	**Other significant^e ^variables**	**Ever Refuse vs.** **Never Refuse**	**Other significant^e ^variables**

Any prescription use of select meds^b^	0.82 (0.47, 1.43)	HMO A, Up-to-date, With ear disorders or respiratory diagnoses.	*	*
Any Inpatient Use^b^	0.73 (0.44, 1.20)	HMO A, With respiratory, skin disorder, or seizure diagnoses.	0.80 (0.40, 1.58)	HMO A, With ear disorder or seizure diagnoses.
Any ER Use^b^	0.81 (0.56, 1.15)	HMO A, Medicaid, With ear disorder, respiratory, skin disorder, or seizure diagnoses.	0.99 (0.59, 1.68)	HMO A, Males, With ear disorder or respiratory diagnoses.
Total # of prescription use of specified med per person per enrollment day^d^	0.93 (0.80, 1.08)	Living in a white majority community, Up-to-date, With ear disorder, respiratory, skin disorder, or seizure diagnoses.	0.96 (0.67, 1.40)	With ear disorder or respiratory diagnoses.
Total # of ER use per person per enrollment day^d^	**0.80 (0.64, 0.997)**^#^	HMO B, Males, Living in a *non-white *majority community, With ear disorder, respiratory, skin disorder, or seizure diagnoses.	1.13 (0.77, 1.66)	HMO B, *Not *up-to-date, With ear disorder diagnoses.
Total # of inpatient days per person^c^	**0.22 (0.09, 0.53)**^#^	HMO B, *Not *up-to-date, *Without *ear disorder diagnosis, With respiratory, skin disorder, and seizure diagnoses.	0.9670 (0.26, 3.54)	With respiratory or seizure diagnoses.
Total # of outpatient visits per person per enrollment day^d^	**0.88 (0.79, 0.98)**^#^	Living in a white majority community, With ear disorder, respiratory, skin disorder, or seizure diagnoses.	0.97 (0.66, 1.41)	With ear disorder or respiratory diagnoses.
Total # of well-child visits per person per enrollment day^d^	1.05 (0.98, 1.12)	HMO A, *Without *ear disorder diagnoses.	0.97 (0.70, 1.35)	HMO A

^a^adjusted for HMO, up-to-date immunization status, gender, Medicaid status, and select high use medical conditions.

^b^Logistic regression model was used.

^c^Poisson regression models with log transformation was used.

^d ^General linear model with log transformation was used.

^e ^P-value < 0.05.

*Due to majority of age-6 group children took antibiotics, asthma, or seizure medications, no adjusted odds ratios were calculated. 86 (93.48%) of 92 ever-refusers in the age 6 group versus 282 (77.90%) of 362 never-refusers took antibiotics, asthma, or seizure medications.

^# ^P-value < .05

Table 5 also compared length of hospitalization, frequency of antibiotics, asthma, or seizure prescriptions, outpatients, well-child and emergency room visits between refusers and non-refusers, controlling for up-to date immunization status, HMO site, gender, Medicaid status, and select high use medical conditions. Although in the age 6 group, refusers and non-refusers were similar, refusers in the age 2 and under group had fewer hospital days per person (P-value = 0.0006), were less frequently seen in outpatient settings (P-value = 0.02) or emergency rooms (P-value = 0.047) than non-refusers.

## Conclusion

Our results indicate that there is poor agreement between refusal status in charts and immunization registries. Policies aimed at educating refusers would be well-served by improvement in immunization registries. Immunization registries possibly may be improved by adding a pull-down field that requires providers to specify a reason for refusal (for example, minor physical illness, major physical illness, allergy, parental refusal, etc). The alternative, tracking children through medical records would be both expensive and time consuming for busy pediatric clinics.

Refusers are more likely to come from well-educated and higher income areas than non- refusers. It should be stressed that this paper makes inference about the socio-economic make-up of the communities, not the specific children themselves. Refusers were found not to have opted out of the health care system and continue, although less frequently for the age 2 and under group, to use services in the health plans. Refusers in the age 2 and under group had fewer hospital days per person, and had fewer outpatient and emergency room visits than non-refusers. The trend toward higher levels of antibiotic use in older refusal children is consistent with higher rates of illness observed in previous epidemiological studies.[27,28]

A previous study found that the consequences of not being immunized can be serious. One retrospective study investigated a cohort of 3 to 18 year old Colorado school children from 1987–1998[1] to see if individuals and communities experienced adverse events from personal exemption to immunization. The study found that children with personal (non-medical) exemptions to vaccines were 22 times more likely to be infected with measles and 5.9 times more likely to be infected with pertussis. They also found that unvaccinated children in day care, who already have an increased susceptibility to disease, are up to 60 times more likely to acquire the disease than their vaccinated peers. It found that refusers seem to be able to transmit disease to vaccinated individuals when the two groups are mixed in a school or during an outbreak. Similarly, another study that looked comprehensively at the health consequences of religious and philosophical exemption from immunization laws[29] in a cohort of 5–19 year olds nation-wide from 1985–1992 found that refusers were 35 times more likely than vaccinated individuals to contract measles from 1985–1992 in the United States. A third study analyzed the relationship between state-level rates of nonmedical exemptions at school entry and pertussis incidence data for individuals aged 18 years or younger.[30] They found that an increased pertussis incidence was associated with an easier granting of exemptions (incidence rate ratio = 1.53; 95% confidence interval, 1.10–2.14) and the availability of personal belief exemptions (incidence rate ratio = 1.48; 95% confidence interval, 1.03–2.13). The decision to refuse immunization must carefully balance individual rights and social responsibility. If a large enough number people decide to forgo immunizations it runs the risk of seeing a resurgence of vaccine preventable diseases, especially measles.[1,2] Numerous reports confirm the probability of outbreaks starting in unvaccinated individuals and then spreading to children whose vaccination may have failed.[1]

Because of the important public health implications involved, it is important to understand characteristics of children who refused to be immunized. Our results show that refusers had not opted out of health care system but continued to participate in it. There are opportunities at regular clinic visits to provide information to refusers to influence vaccine attitudes. Our findings suggest that interventions may be implemented at clinics to improve immunization compliance and long-term follow-up with this subgroup of children. Since parents of refusers are from well educated communities, interventions should target these communities to communicate the most up-to-date research on vaccine adverse events and the consequences of vaccine preventable diseases.[31] While the parents may not permit all routine immunizations, some may be given. Progress may be made if the parents are given a wide amount of information about vaccinations including: which diseases are not affected by herd immunity, more information about the diseases, another viewpoint about vaccine side effects, and information that all vaccines routinely given to children, except some influenza vaccines, are thimerosol free. [32-36]

Our study has several limitations. Use of geocoding to assign the race, education, and household income level of the child provides information about the communities in which the child lives, and may result in misclassification of the specific child. However, census-based data on income and education at the block group, tract, and ZIP code level have been widely used as proxies for the SES of individuals in studies of health outcomes and health funds allocation[4-20,22-24]. Census-based SES data at the block group and tract level have also been used to characterize community socioeconomic conditions [17-20]. We have based our interpretation of the results on the characteristics of communities in which children live. Due to the possibility of misclassification, care should be taken when generalizing these results to the child level. Second, due to the small total number of refusers, as well as the fact that some providers use refusal and medical contraindication interchangeably, and that some providers tend to always use "ever refusal," we documented "refusal" as either "contraindication" or "refusal." Vaccine refusals include true vaccine refusals, true contraindications, as well as deferrals for minor illnesses. Combining these three groups is a limitation of this study, but it is unlikely that the data requested could be accurately obtained without undertaking an expensive prospective study that includes direct observation of provider-patient interactions. Third, refusers may be more likely to receive care from alternative providers, such as naturopathic physicians. Since alternative providers are not a part of the HMO system and few health insurance policies pay for these services, we do not have access to data on care provided by alternative providers. Fourth, due to the small total number of refusers in the age 6 group, a continuous enrollment was not required and "per person per day" analysis strategy was adopted.

The present report found that refusers, have slightly lower but similar health care utilization in the short-run except for prescriptions in two year olds. Thus, these families have not opted out of health care completely, and practitioners will have numerous opportunities to counsel these parents in the first two years of their children's lives, which is consistent with AAP policy statement on vaccine refusers.[37]

## Competing interests

The authors declare that they have no competing interests.

## Authors' contributions

FW Concept and design; acquisition of data; analysis and interpretation of data; drafting of the manuscript; critical revision of the manuscript for important intellectual content; statistical analysis; supervision. JM Concept and design; acquisition of data; analysis and interpretation of data; drafting of the manuscript; critical revision of the manuscript for important intellectual content; statistical analysis; supervision. MG Concept and design; analysis and interpretation of data; critical revision of the manuscript for important intellectual content. MCM Concept and design; analysis and interpretation of data; critical revision of the manuscript for important intellectual content. AMH Concept and design; acquisition of data; analysis and interpretation of data. BC Acquisition of data; analysis and interpretation of data; administrative, technical or logistical support. JDN Concept and design; analysis and interpretation of data; critical revision of the manuscript for important intellectual content. All authors have read and approved the final manuscript.

## Pre-publication history

The pre-publication history for this paper can be accessed here:


